# Transcriptomic and Metabolomic Analyses Reveal Key Metabolites, Pathways and Candidate Genes in *Sophora davidii* (Franch.) Skeels Seedlings Under Drought Stress

**DOI:** 10.3389/fpls.2022.785702

**Published:** 2022-03-02

**Authors:** Xin Zhao, Li-Juan Huang, Xiao-Fu Sun, Li-Li Zhao, Pu-Chang Wang

**Affiliations:** ^1^College of Animal Science, Guizhou University, Guiyang, China; ^2^Guizhou Institute of Prataculture, Guiyang, China

**Keywords:** *Sophora davidii*, transcriptomics, metabolomics, drought stress, physio-biochemical characteristics

## Abstract

Soil aridification and desertification are particularly prominent in China’s karst areas, severely limiting crop yields and vegetation restoration. Therefore, it is very important to identify naturally drought-tolerant plant species. *Sophora davidii* (Franch.) Skeels is resistant to drought and soil infertility, is deeply rooted and is an excellent plant material for soil and water conservation. We studied the transcriptomic and metabolomic changes in *S. davidii* in response to drought stress (CK, control; LD, mild drought stress; MD, moderate drought stress; and SD, severe drought stress). *Sophora davidii* grew normally under LD and MD stress but was inhibited under SD stress; the malondialdehyde (MDA), hydrogen peroxide (H_2_O_2_), soluble sugar, proline, chlorophyll a, chlorophyll b and carotenoid contents and ascorbate peroxidase (APX) activity significantly increased, while the superoxide dismutase (SOD), peroxidase (POD) and catalase (CAT) activities and soluble protein content significantly decreased. In the LD/CK, MD/CK and SD/CK comparison groups, there were 318, 734 and 1779 DEGs, respectively, and 100, 168 and 281 differentially accumulated metabolites, respectively. Combined analysis of the transcriptomic and metabolomic data revealed the metabolic regulation of *S. davidii* in response to drought stress. First, key candidate genes such as *PRR7*, *PRR5*, *GI*, *ELF3*, *PsbQ*, *PsaK*, *INV*, *AMY*, *E2.4.1.13*, *E3.2.1.2*, *NCED*, *PP2C*, *PYL*, *ABF*, *WRKY33*, *P5CS*, *PRODH*, *AOC3*, *HPD*, *GPX*, *GST*, *CAT* and *SOD1* may govern the drought resistance of *S. davidii*. Second, three metabolites (oxidised glutathione, abscisic acid and phenylalanine) were found to be related to drought tolerance. Third, several key candidate genes and metabolites involved in 10 metabolic pathways were identified, indicating that these metabolic pathways play an important role in the response to drought in *S. davidii* and possibly other plant species.

## Introduction

Drought stress is one of the main stress factors affecting normal plant growth and development (You et al., 2019). The reduction in crop yield caused by drought exceeds the total reduction in yield caused by other environmental stresses (Abdou et al., 2021). As a result, drought has become a bottleneck in the development of agricultural production and ecological restoration in many regions (Ali et al., 2021). Under the increasingly severe threat of drought worldwide, the adaptation of plants to drought stress and their drought resistance mechanisms have become popular research topics in many fields, such as environmental science, ecology and genetics (Rahimi et al., 2019; Wan et al., 2022).

Plants adapt to drought through a series of morphological, physiological, biochemical, metabolic and molecular changes when water is lacking (Baozhu et al., 2022; Thanmalagan et al., 2022). Plant species such as soybean can reduce the damage caused by water loss by increasing the contents of endogenous hormones, inhibiting photosynthesis, altering their morphology and structure, slowing their growth and reducing their biomass (Ramirez-Villegas et al., 2018; Du et al., 2020). Plant species such as safflower can also resist drought by altering the physiological and biochemical metabolism of cells, such as by increasing the content of cell-permeable substances to maintain cell turgor and increase cell hydrophilicity and cell membrane permeability (Bo et al., 2020), activating endogenous protection systems to increase the activity of protective enzymes such as antioxidant-related enzymes and nonenzymatic antioxidants, maintaining the balance of active oxygen metabolism throughout the plant and resisting oxidative membrane damage (Ahanger and Agarwal, 2017).

Plants resistance to drought increases through morphological or cell physiological and biochemical changes. However, these morphological or physiological changes are the result of changes in related metabolites and gene expression in plants. Therefore, exploring the molecular mechanism underlying plant drought resistance and understanding the drought resistance mechanism are necessary for improving plant drought resistance and breeding efforts. In recent years, many important advances have been made in transcriptomic and metabolomic research. Liu et al. (2022) found that several transcription factors (TFs), including members of the WRKY, AP2/ERF, C2H2, bHLH, MYB, bZIP, NAC, LEA, MADS, and GRAS families, are differentially expressed in *Seriphidium transiliense* seedlings in response to drought stress. Sun et al. (2022) found DEGs were involved in tryptophan and α-linolenic acid metabolism, flavonoid and phenylpropanoid biosynthesis and the mitogen-activated protein kinase (MAPK) signalling pathway in *Tamarix taklamakanensis* seedlings in response to drought stress. In addition, in-depth transcriptomic analyses of *Brassica napus* (Xiong et al., 2018), durum wheat (Gullì et al., 2022), sweet sorghum (Wang et al., 2022) and *Agrostis stolonifera* (Ma et al., 2017) under drought stress were performed, and the corresponding drought stress-responsive genes were identified. The volatile substances released by plants through secondary metabolism are also important means by which plants resist adverse conditions. Bo et al. (2020) found that three metabolites in safflower (galactitol, neoxanthin and arbutin) were related to drought tolerance, and the regulatory mechanism differed among genotypes. Zhao et al. (2021b) analysed the metabolome of chicory and found that the mechanism of drought tolerance involved glycolysis, phenol metabolism, the tricarboxylic acid cycle, glutamate-mediated proline synthesis, amino acid metabolism and unsaturated fatty acid synthesis. Metabolites can also accumulate in response to drought stress; researchers have identified the key metabolic pathways and metabolites in *Glycyrrhiza uralensis* (Zhang et al., 2022), rice (Auler et al., 2021), tobacco and soybean (Rabara et al., 2017) in response to drought stress.

The karst area in Guizhou Province, China, is one of the most typical, complex and largest areas of karst development in China and worldwide. The karst distribution area accounts for 85% of the area of the province. The unique soil formation background and climatic conditions of the carbonate parent rock in this area have caused problems such as slow soil formation, a shallow soil layer, a low organic matter content, poor water and soil conservation capacity and severe rocky desertification. The province’s average annual total precipitation is relatively high, but precipitation quickly infiltrates into the ground. After a few days of high temperature and sunny days, the soil water content (SWC) decreases, and temporary drought effects events occur on the surface. Therefore, plants in karst areas generally have drought tolerance and other adaptations to rocky soil conditions. Shrubs are the most common vegetation type in south-western karst areas, among which *Sophora davidii* (Franch.) Skeels, a leguminous shrub of the genus *Sophora*, is an important dominant species. Because of its advantages, such as drought tolerance, tolerance to infertility, strong adaptability and rich nutritional and medicinal value, *S. davidii* is cultivated as an important plant resource for karst ecological environment management, forage farm establishment and soil improvement (Yang et al., 2019; Gao et al., 2020; Ma et al., 2020; Zhao et al., 2021a). At present, research on *S. davidii* drought stress has focused mainly on the morphological structure and physiological and biochemical responses of this species (Zhao et al., 2017; Zhang et al., 2020), but the underlying molecular mechanisms are still unclear. Thus, to explore the changes in *S. davidii* under drought stress and identify the important mechanisms through which this species responds to stress, this experiment used *S. davidii* as a research object and involved subjecting potted seedlings to drought treatment, after which their leaves were subjected to transcriptomic and metabolomic analysis. RNA sequencing (RNA-seq) revealed core drought resistance candidate genes in young leaves of *S. davidii* under drought stress, and LC/MS was used to conduct metabolomic analysis to understand the drought-responsive metabolites of *S. davidii*. The integration of transcriptomics and metabolomics data provides a theoretical reference for the growth of *S. davidii* under drought conditions and provides theoretical support for genetic engineering to breed highly drought-resistant *S. davidii* varieties.

## Materials and Methods

### Plant Materials and Experimental Design

*Sophora davidii* seeds were obtained from the Grassland Science Laboratory of the College of Animal Science of Guizhou University of China and planted in plastic pots (43 cm in length × 21 cm in width × 14 cm in height; approximately 30 plants per pot) at Guizhou University (26°27'N, 106°39'E), Huaxi district, Guiyang, Guizhou Province, China. *Sophora davidii* is widely grown in Southwest China (Zhao et al., 2021a). Nutrient-rich soil that included clay was mixed at a 1:1 ratio. A moisture meter (Wenzhou Weidu Electronic Co., Ltd., China) combined with the oven drying method was used to measure the SWC at different points, and the results showed good consistency. Drought gradients were established according to the Standard of Classification for Drought Severity SL424-2008 in China (Yan et al., 2013). The highest SWC was equal to the pots being fully watered (FW). According to the drought severity classification, the soil water gradients were established as follows: control (CK), 75%–80% of FW conditions; mild drought (LD), 55%–60% of FW conditions; moderate drought (MD), 40%–45% of FW conditions; and severe drought (SD), 30%–35% of FW conditions. Three pots were included for each SWC treatment. The experiment began on 27 September 2020. *Sophora davidii* seeds of uniform size were sown into plastic pots. The soils were then regularly watered, and the plants were allowed to grow for 2 months under natural conditions, after which drought conditions were applied. When each treatment reached its corresponding SWC (consistent with the levels corresponding to those of the CK, LD, MD and SD treatments), all the SWC treatments were maintained for 12 days. The soils were checked daily, and the SWCs were adjusted accordingly. All leaf samples were collected between 9:00 and 11:00 AM. on the morning of December 9, 2020, which was sunny. The physiological and biochemical characteristics of *S. davidii* leaves were measured in triplicate. Three replicate leaf samples and six replicate leaf samples were collected for transcriptome and metabolomic analyses, respectively. The leaf samples were frozen immediately in liquid nitrogen for subsequent experiments.

### Measurements of Physio-Biochemical Characteristics

#### Relative Water Content and Morphological Characteristic Measurements

The relative water content (RWC) of leaves = (FWL − DW)/(TW − DW) × 100, where FWL represents the fresh weight of the leaf sample, TW represents the weight of the leaf after it was immersed in distilled water in the dark for 24 h and DW represents the dry weight after heating at 65°C for 24 h.

The growth rate of plant height = (before treatment plant height—after treatment plant height)/12. The absolute plant height (the vertical height from the ground to the top of the plant) was measured before and after treatment. The RWC and growth rate were measured for 10 plants in each treatment.

Leaf samples were imaged with an Epson Perfection V800 Photo Scanner. The leaf parameters, including leaf width, leaf length, and leaf area, were analysed using WinFOLIA software (Regent Instruments, Inc., Canada). The fresh seedlings were divided into aerial parts and root systems, and then, both aerial parts and roots were dried at 105°C for 1 h and then at 80°C for 72 h followed by weighing.

#### MDA and H_2_O_2_ Content Measurements

The lipid peroxidation level in terms of malondialdehyde (MDA) content was determined by the method described by Cakmak and Horst (1991). Fresh leaves (0.5 g) were ground in 10 ml of trichloroacetic acid (TCA; 0.1%) using a mortar and pestle. Then, 4 ml of 0.5% TBA was added to 1 ml of the supernatant. The mixture was heated, cooled and then centrifuged at 10,000 × *g* for 5 min. The absorbance was read at 532 nm. The MDA content was calculated using an extinction coefficient of 155 mM^−1^ cm^−1^. To measure H_2_O_2_ content, fresh samples (0.2 g) were extracted with 5 ml of 0.1% TCA (w/v), placed in an ice bath and then centrifuged at 12,000 × *g* for 15 min at 4°C (Velikova et al., 2000). Afterwards, 0.5 ml of 100 mM phosphate buffer (pH 7.0) and 1 ml of 1 M potassium iodide were added to 0.5 ml of the supernatant. The absorbance was read at 390 nm, and a standard curve was used to calculate the H_2_O_2_ content.

#### Measurement of Enzyme Activities

Weigh 0.3 g of plant sample, add 5 ml of prechilled 50 mM PH7.8 sodium phosphate buffer (containing 1 mM EDTA^.^Na_2_ and 2% PVP), grind into a homogenate in an ice bath and centrifuge at 4°C for 15 min at 12,000 rpm, the supernatant is the crude enzyme solution for measuring superoxide dismutase (SOD), peroxidase (POD), catalase (CAT) and ascorbate peroxidase (APX) activities.

SOD (EC 1.15.1.1) activity determination was performed according to the methods of Tandy et al. (1989). A 3 ml reaction system contains 1.5 ml of 50 mM sodium phosphate buffer (pH7.8), 0.25 ml of distilled water, 0.3 ml of 130 mM methionine (Met), 0.3 ml of 750 μM nitro tetrazolium blue chloride (NBT), 0.3 ml of 100 μM EDTA.Na_2_, 0.3 ml of 20 μM riboflavin and 0.05 ml of enzyme solution. Three tubes were prepared simultaneously and two control tubes were prepared with sodium phosphate buffer instead of enzyme solution. After mixing, one control tube was placed in the dark and the other two tubes were reacted at 25°C under 300 μmol m^−2^ s^−1^ light for 5 min. The absorbance values of the other two tubes at 560 nm were measured using the dark treatment tube as the zeroing tube, and 50% inhibition of the photochemical reaction was taken as one unit of enzyme activity.

POD (EC 1.11.1.7) activity was determined according to the methods of (Machly and Chance, 1955). Add guaiacol and 30% H_2_O_2_ to 50 mM PH7.8 sodium phosphate buffer and dissolve with stirring to make a reaction mix. In the control tube, add 3 ml of reaction mix and 0.1 ml of sodium phosphate buffer, and in the sample tube, add 3 ml of reaction mix and 0.1 ml of enzyme solution, and immediately time the reading of absorbance value at 470 nm, each reading was taken once at 1 min, and the total reading was 5 min. A change in absorbance value of 0.01 in 1 min was taken as 1 unit of enzyme activity.

CAT (EC 1.11.1.6) activity determination was performed according to the methods of Mchale (1987). A 3 ml reaction system contains 1.5 ml of 50 mM sodium phosphate buffer (pH 7.8), I ml of distilled water, 0.2 ml of enzyme solution and 0.3 ml of 0.1 mM H_2_O_2_ and control tube 0.3 ml distilled water instead of H_2_O_2_. The reaction was started by adding H_2_O_2_ to the sample cup and immediately timing the reaction and reading the absorbance value at 240 nm at 1 min intervals. A total of 3 min was taken and the amount of enzyme that decreased by 0.1 in absorbance in 1 min was taken as 1 unit of enzyme activity.

Ascorbate peroxidase (APX, EC 1.11.1.11) activity was determined according to the methods of Murshed et al. (2008). A 3 ml reaction system contained as: 1.8 ml of 50 mM sodium phosphate buffer (pH 7.8), 0.1 ml of 15 mM ascorbic acid, 1 ml of 0.3 mM H_2_O_2_ and 0.1 ml of enzyme solution, the control tube was replaced with 0.1 ml of buffer. The reaction was initiated by adding the enzyme solution to the sample cup, immediately timed and the absorbance values were read at 290 mm, with each 1 min reading being taken once for a total of 4 min. A change in absorbance value of 0.01 in 1 min was taken as 1 unit of enzyme activity.

#### Measurement of Photosynthetic Pigments

The estimation of photosynthetic pigments was performed according to the methods of Niroula et al. (2021). Fresh leaves (100 mg) were cut into small pieces, and 10 ml of dimethyl sulfoxide (DMSO) was added. The test tubes were incubated at 45°C for 40 min, and the absorbance was read at 645 and 663 nm for chlorophyll and at 480 and 510 nm for carotenoid contents.

#### Measurement of the Contents of Osmotic Adjustment Substances

Soluble protein content was estimated in each sample according to the methods of Bradford (1976) using the coomassie brilliant blue G250 dye. A sample of 0.2 g leaves was ground and centrifuged at 8,000 × *g*. Dye solution was added to the supernatant, and the absorbance was read at 595 nm for no more than 30 min.

The proline content was determined by the acid indene triketone method (Kant et al., 2006). Fresh leaves (0.3 g) were homogenised in 3% sulfosalicylic acid. The filtrate was reacted in response to 1 ml each of glacial acetic acid and acid ninhydrin in a test tube kept in a water bath at 100°C for 1 h. The reaction was terminated by placing the test tube on ice. The absorbance of the mixture was read at 520 nm.

The soluble sugar content was determined by anthrone colorimetry (Jan and Roel, 1993). A mass of 0.2 g of the dried sample with finely ground leaves was placed into a 10 ml centrifuge tube, extracted with 80% ethanol, centrifuged and then diluted to 25 ml. The ethanol extract of sugar was placed into a 2 ml water bath to distil the ethanol, 10 ml of water was added and the mixture was stirred until the sugar was completely dissolved. Two millilitres of the supernatant was pipetted into a test tube, the OD value at 620 nm was recorded and the soluble sugar content was calculated.

### Statistical Analysis

The data were collated using WPS office software and SPSS 25.0 software for one-way ANOVA statistical analysis and the least significant difference (LSD) method for multiple comparisons, with significant differences defined at *p* < 0.05. All data are the means ± standard errors of three replicates, and SigmaPlot 14.0 was used for graphical plotting.

### Metabolomic Profiling

Widely targeted metabolomic profiling was carried out by a commercial service company (Novogene, Beijing, China). In brief, 100 mg of leaves [24 samples (four treatments [CK, LD, MD, SD]) × six biological replicates] was individually ground in liquid nitrogen, and the homogenates were resuspended in prechilled 80% methanol and 0.1% formic acid thorough vortexing. The samples were incubated on ice for 5 min and then centrifuged at 15,000 × *g* at 4°C for 20 min. A portion of the supernatant was diluted with LC–MS-grade water such that the final constitution was equal to 53% methanol. The samples were subsequently transferred to a new Eppendorf tube and then centrifuged again at 15,000 × *g* at 4°C for 20 min. Finally, the supernatant was injected into the HPLC–MS/MS system, and analysis was performed using a Vanquish UHPLC system (Thermo Fisher, Germany) equipped with an Orbitrap Q Exactive™ HF-X mass spectrometer (Thermo Fisher, Germany; Want et al., 2013). The HPLC and MS conditions were the same as those used by Chen et al. (2013). Compound Discoverer 3.1 (CD 3.1; Thermo Fisher) was used for peak alignment, peak picking and quantitation of each metabolite. The metabolites were subsequently annotated using the Kyoto Encyclopedia of Genes and Genomes (KEGG) database.^1^ Principal component analysis (PCA) and partial least squares discriminant analysis (PLS-DA) were performed with metaX (dynamic and comprehensive software for processing metabolomic data). We applied univariate analysis (*t*-test) to calculate the statistical significance (value of *p*). Metabolites with a variable importance in projection (VIP) > 1, a *p* < 0.05 and a fold-change (FC) ≥ 2 or ≤ 0.5 were considered to be differentially accumulated.

### Transcriptome Sequencing and Gene Expression Profiling

Four leaf samples from each SWC treatment were used for RNA-seq. Total RNA was extracted from each sample using TRIzol reagent (Invitrogen, United States). After RNA quality assessment, mRNA purification and mRNA fragmentation, a cDNA library was constructed, and raw data were obtained by Illumina paired-end sequencing on an Illumina HiSeq 4,000 platform. Clean reads were obtained by removing reads containing adapters, reads containing poly-N sequences and reads of low quality from the raw data. Gene expression levels in each sample were estimated by RSEM (v1.2.15). The clean data were mapped back into the assembled transcriptome using RSEM. The read counts for each sequence were then obtained from the mapping results by Bowtie2 (with a mismatch of 0) and normalised to reads per kilobase of exon model per million mapped reads (RPKM; Li and Dewey, 2011). Differential expression analysis of two conditions/groups was performed using the DESeq R package (1.10.1). The resulting *p* values were adjusted using Benjamini and Hochberg’s approach to control the false discovery rate. The p value was adjusted using the Q value, with Q value < 0.005 and |log2 > 1| set as the thresholds for significant differences in gene expression. Correlation analysis and PCA were used to test the reliability of the samples. Differentially expressed genes (DEGs) were identified in the LD/CK, MD/CK and SD/CK comparison groups. For functional analysis, all the DEGs were subjected to Gene Ontology (GO) annotation^2^ and KEGG^3^ pathway enrichment analyses. The GO and KEGG pathway enrichment results were considered significant when values of *p* adjusted *via* Bonferroni correction (Q values) were ≤ 0.05, and WEGO software was used for statistical analysis of the GO functional categories and KEGG pathway enrichment results.

### Quantitative Real-Time-PCR Validation of DEGs According to Transcriptomic Data

Eighteen DEGs were selected to validate the RNA-seq results. cDNA was synthesised from the same RNA samples used for transcriptome sequencing. We performed quantitative real-time (qRT)–PCR on a CFX Connect™ Real-Time System (Applied Biosystems) together with UltraSYBR mixture (CWBiotech). The parameters of the thermocycler were 95°C for 10 min, followed by 40 cycles of 95°C for 15 s and 60°C for 1 min in a 20 μl reaction mixture. The *Lupinus angustifolius* actin gene was selected as an internal standard for normalisation.

## Results

### Estimation of Related Morphological and Physio-Biochemical Traits of *Sophora davidii*

**Figure 1:**
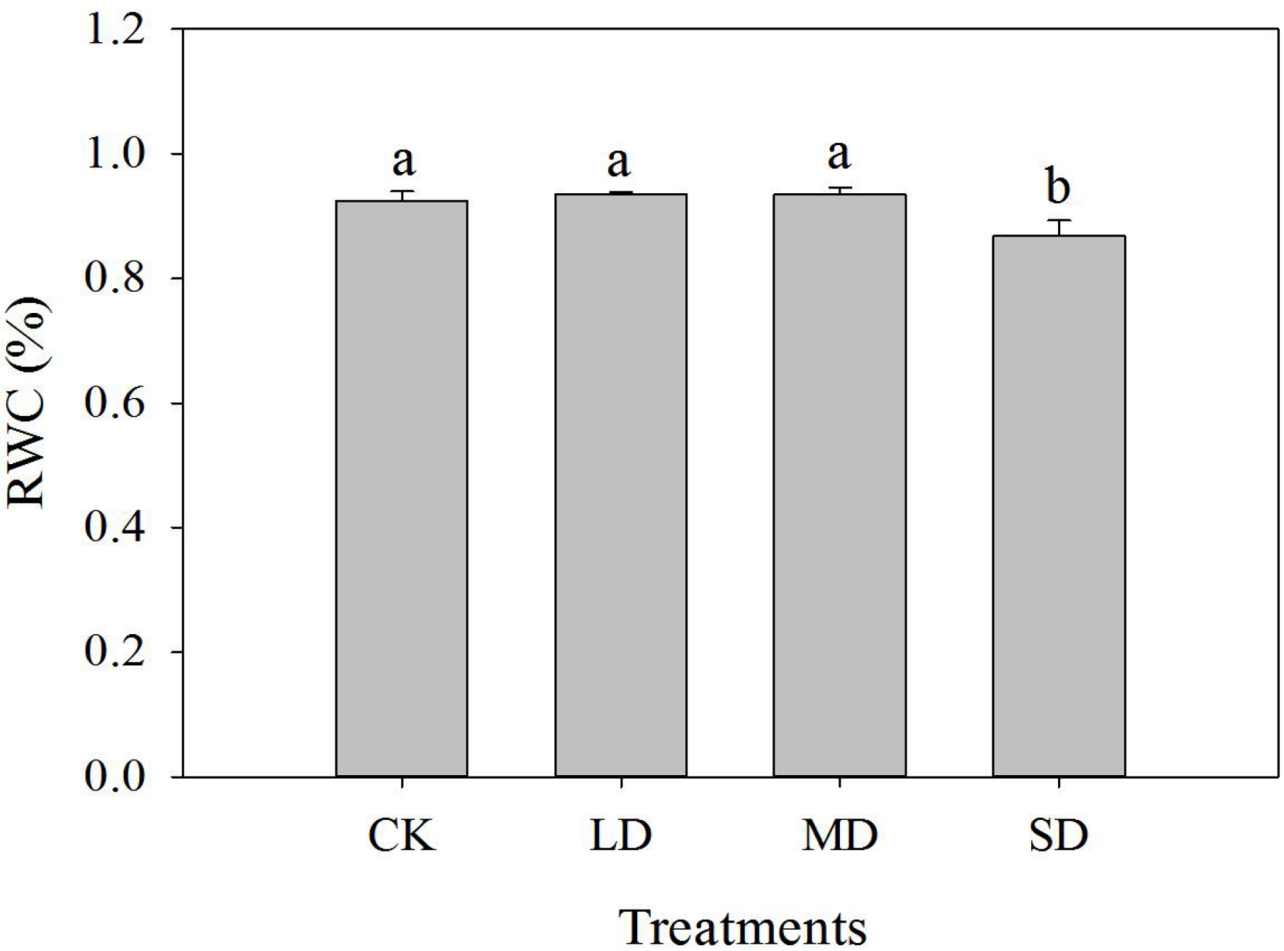
Effects of drought stress on the relative water content (RWC) of *Sophora davidii* leaves. The different lowercase letters indicate significant differences (*p* < 0.05) for each index among the different drought treatments.

The leaf RWC of *S. davidii* did not differ significantly between the LD, MD and CK treatments (Figure 1). The leaf RWC of *S. davidii* under SD stress obviously decreased compared with that under the CK treatment (*p* < 0.05). Interestingly, although the SWC of *S. davidii* decreased to 30% (SD stress), the leaf RWC remained relatively high.

Compared with that of the CK, the rate of increase of *S. davidii* plant height increased under MD stress but decreased under LD and SD stress. The dry weight of the aboveground parts increased under LD and MD stress but decreased under SD stress and peaked under MD stress, but the difference was not significant. The dry weight of the roots increased under drought stress and increased significantly under LD stress (*p* < 0.05). The leaf length, leaf width and leaf area of *S. davidii* under LD, MD and SD stress were lower than those of the CK, and they were all significantly reduced under SD stress (*p* < 0.05, Supplementary Figure S1).

**Figure 2:**
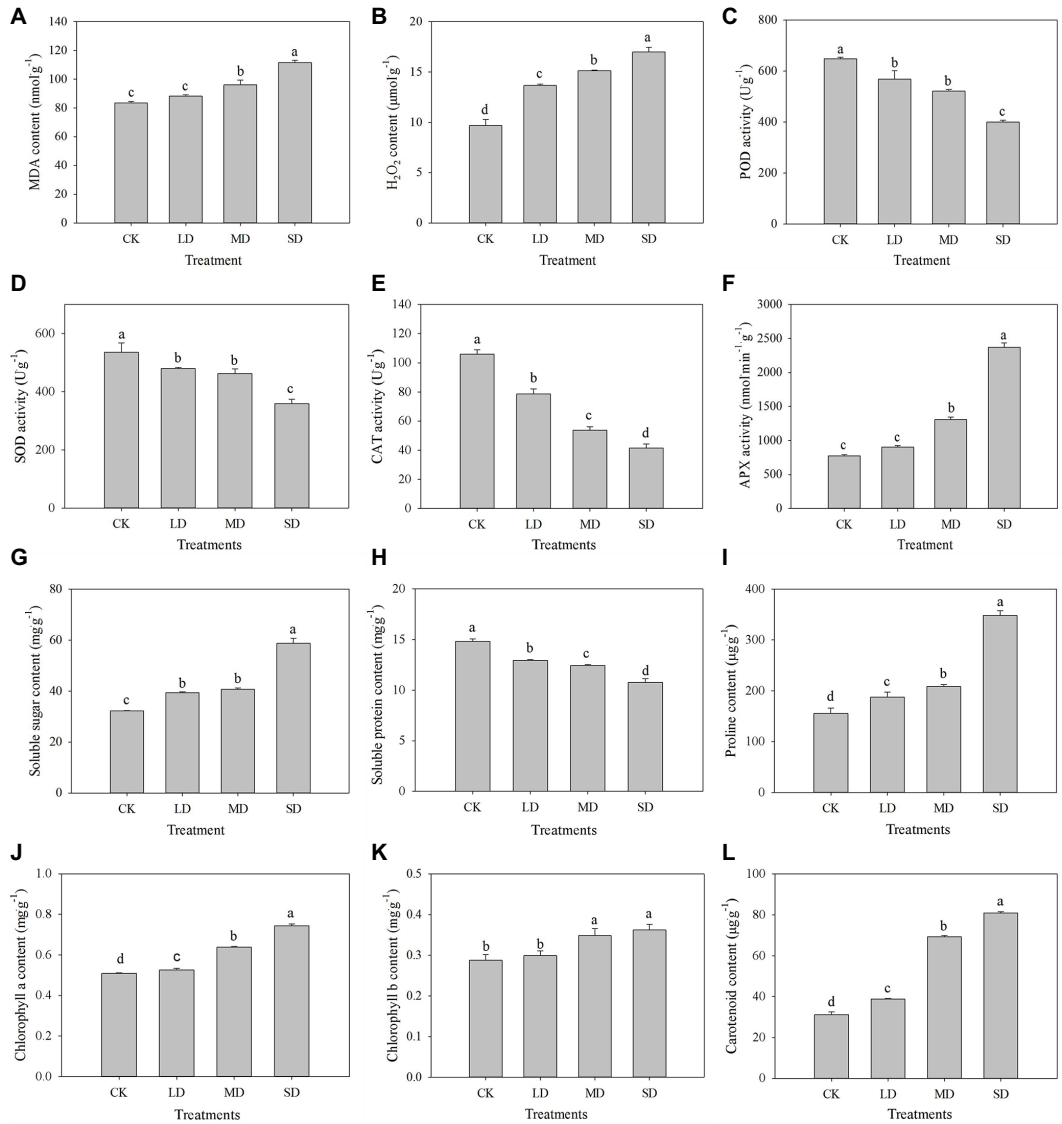
Physio-biochemical characteristics change in *S. davidii* under different drought treatment conditions. Effects of drought stress on **(A)** MDA content, **(B)** H_2_O_2_ content, **(C)** POD activity, **(D)** SOD activity, **(E)** CAT activity, **(F)** APX activity, **(G)** soluble sugar content, **(H)** soluble protein, **(I)** proline content, **(J)** chlorophyll a content, **(K)** chlorophyll b content and **(L)** carotenoid content. The different lowercase letters indicate significant differences (*p* < 0.05) for each index among the different drought treatments.

The MDA, H_2_O_2_, soluble sugar, proline, chlorophyll a, chlorophyll b and carotenoid contents and APX activity of *S. davidii* increased with increasing drought stress (Figure 2). Compared with those under the CK treatment, H_2_O_2_, soluble sugar, proline, chlorophyll a and carotenoid contents under LD, MD and SD stress significantly increased (*p* < 0.05). Moreover, the MDA and chlorophyll b contents and APX activity significantly increased under MD and SD stress (*p* < 0.05). However, the POD, SOD and CAT activities and soluble protein content in *S. davidii* significantly decreased with increasing drought stress (Figure 2). Compared with those under the CK treatment, POD, SOD and CAT activities and soluble protein content under LD, MD and SD stress significantly decreased (*p* < 0.05).

### Differentially Accumulated Metabolites Detected in *Sophora davidii*

PCA was used to analyse the dynamic changes in *S. davidii* under drought stress. As shown in Supplementary Figure S2, some plots representing samples of *S. davidii* under the CK, LD, MD and SD treatments clustered together. Other samples showed distinct separation, suggesting significant changes in metabolites in those samples.

**Figure 3:**
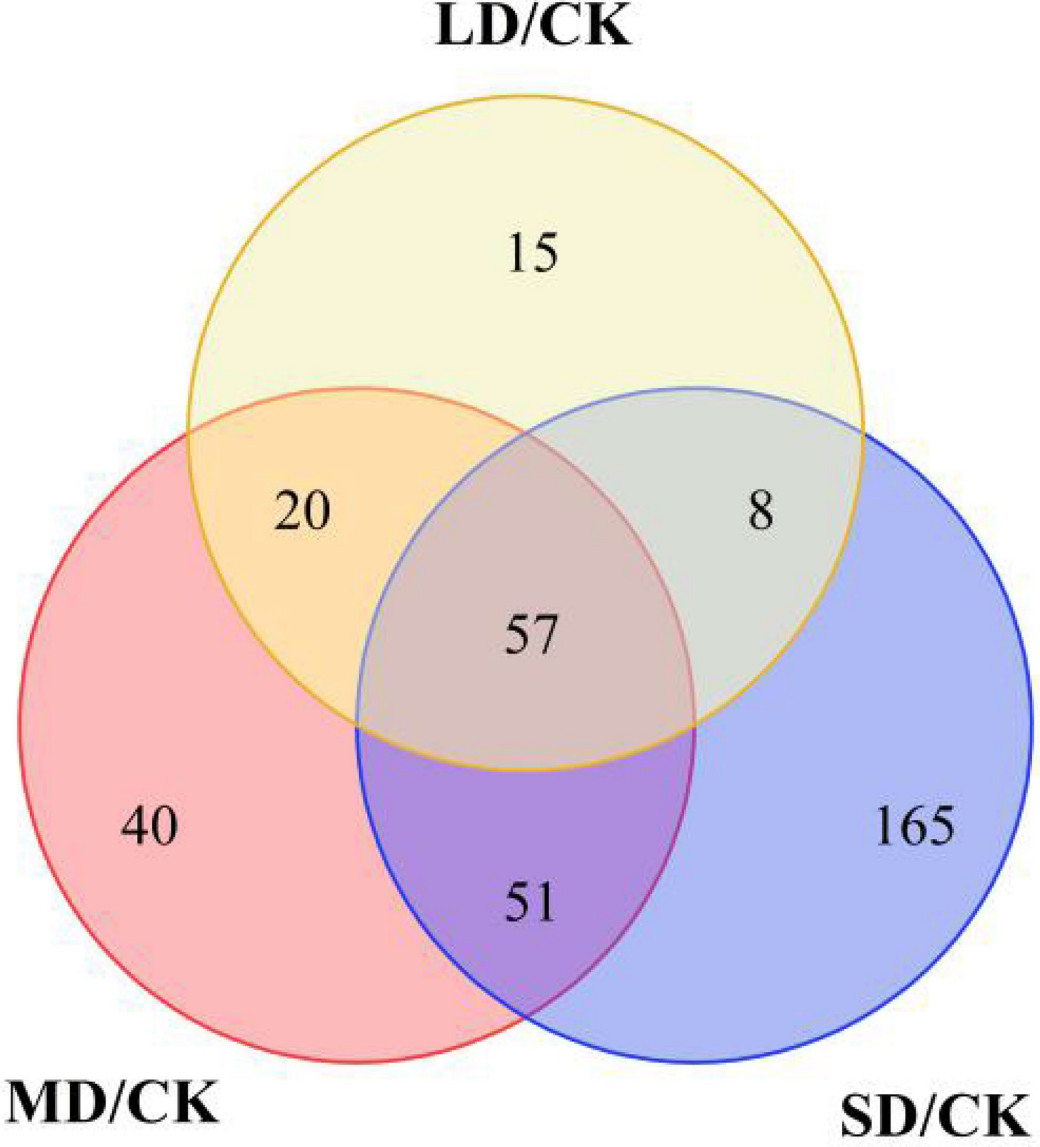
Differentially accumulated metabolites shown in Venn diagram form.

**Figure 4:**
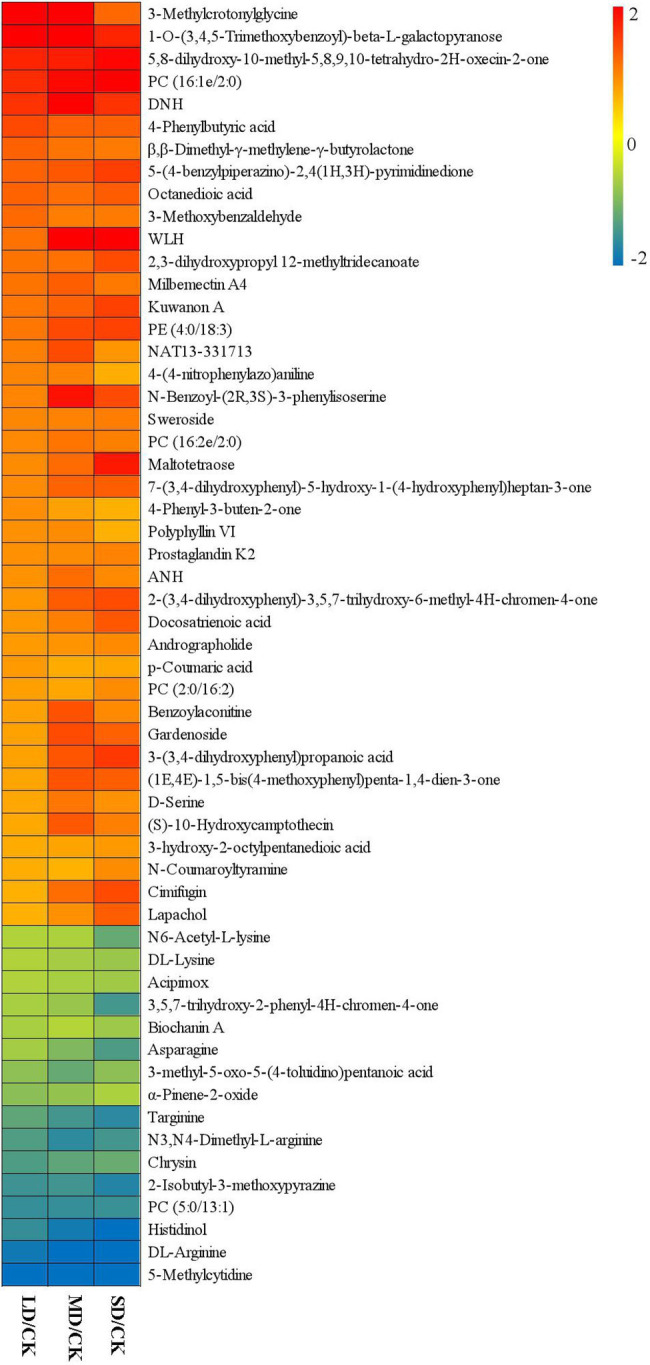
Fold-changes (FCs) of 57 common differentially accumulated metabolites in the four treatment groups. Green indicates downregulated expression, and red indicates upregulated expression.

To determine the metabolites that differentially accumulated in response to drought stress, nonobjective metabolic spectral analysis was carried out *via* LC–MS. Metabolites with a VIP value > 1 and a *p* < 0.05 were considered significantly differentially accumulated metabolites. In total, 306 differentially accumulated metabolites were identified as overlapping according to a Venn diagram (Figure 3). For LD/CK, 100 metabolites significantly differed (Figure 3; Supplementary Table S1); for MD/CK, 168 metabolites significantly differed (Figure 3; Supplementary Table S1); and for SD/CK, 281 metabolites significantly differed (Figure 3; Supplementary Table S1). Among these differentially accumulated metabolites, 57 were common in all three comparison groups (LD/CK, MD/CK and SD/CK; Figures 3, 4).

To identify drought stress-related metabolites in *S. davidii* leaves and determine their increasing/increasing trends under drought stress and water-sufficient conditions, we considered the 57 differentially accumulated metabolites in *S. davidii* common in the three comparison groups to be drought stress-associated metabolites (Figure 4). We found that the contents of 41 metabolites increased and the contents of 16 metabolites decreased in the three comparison groups under drought stress. For example, the contents of β,β-dimethyl-γ-methylene-γ-butyrolactone, 4-phenyl-3-buten-2-one, 3-(3,4-dihydroxyphenyl)propanoic acid and 3-hydroxy-2-octylpentanedioic acid increased in the three groups under drought stress. However, the contents of β,β-dimethyl-γ-methylene-γ-butyrolactone and 4-phenyl-3-buten-2-one decreased with increasing drought stress, and the contents of 3-(3,4-dihydroxyphenyl)propanoic acid and 3-hydroxy-2-octylpentanedioic acid increased with increasing drought stress. The contents of α-pinene-2-oxide, chrysin, asparagine and DL-arginine decreased in the three comparison groups under drought stress. However, the contents of α-pinene-2-oxide and chrysin increased with increasing drought stress, but the contents of asparagine and DL-arginine decreased.

To further analyse the differentially accumulated metabolites related to increased *S. davidii* drought tolerance, KEGG pathway analysis was carried out for all differentially accumulated metabolites in *S. davidii*. KEGG analysis assigned 51 differentially accumulated metabolites to 51 metabolic pathways (Supplementary Table S2). L-Aspartic acid and L-phenylalanine were involved in most KEGG pathways (15 and 12 KEGG pathways, respectively; Supplementary Table S2). In addition, KEGG pathway analysis indicated that 31, 2 and 6 differentially accumulated metabolites were involved in metabolic pathways, phenylpropanoid biosynthesis and amino acid biosynthesis, respectively (Supplementary Table S2). In particular, five differentially accumulated metabolites (D-serine, N6-acetyl-L-lysine, biochanin A, p-coumaric acid and glycerol chrysin) were found to be involved in these KEGG pathways among the three comparison groups of *S. davidii* under drought stress (Supplementary Table S3).

### DEGs Identified in *Sophora davidii*

**Figure 5:**
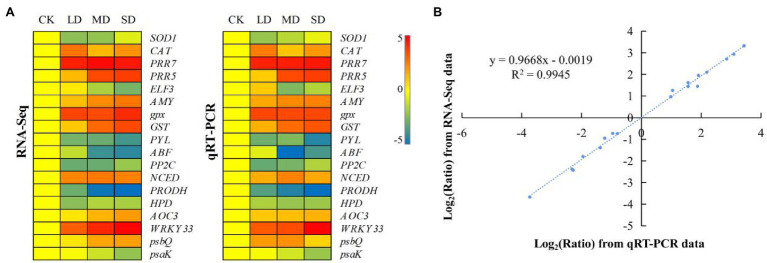
Expression patterns of 18 selected genes identified by RNA-seq were verified *via* quantitative real-time (qRT) PCR. **(A)** Heatmap showing the expression changes [log_2_(fold-change [FC])] in response to the CK to SD treatments for each candidate gene, as measured by RNA sequencing (RNA-seq) and qRT–PCR. **(B)** Scatter plot showing the changes in the expression [log_2_(FC)] of selected genes based on RNA-seq *via* qRT–PCR. The gene expression levels are indicated by coloured bars.

The same materials used in the metabolomic analysis were sequenced, yielding 85.9 Gbp of raw data, with each sample ranging from 6.67 to 8.03 Gbp (Supplementary Table S4; the data were deposited in the NCBI Sequence Read Archive (SRA) under accession PRJNA750237). A total of 18 DEGs were randomly selected for qRT–PCR validation (Supplementary Table S5; Figure 5). The results showed that their expression patterns were highly correlated with the fragments per kilobase per million reads (FPKM) values of the RNA-seq data, corroborating the reliability of the transcriptomic data.

PCA revealed strong correlations between replicates within each group and weaker correlations between different treatment groups, with the weakest correlation occurring between the CK and MD treatment groups (Supplementary Figure S3a). PCA confirmed the reliability and separation of the samples, in which PC1 explained 18.2% of the total variance and PC2 explained 15.8%. The CK and MD treatments showed the greatest separation between groups (Supplementary Figure S3b).

**Figure 6:**
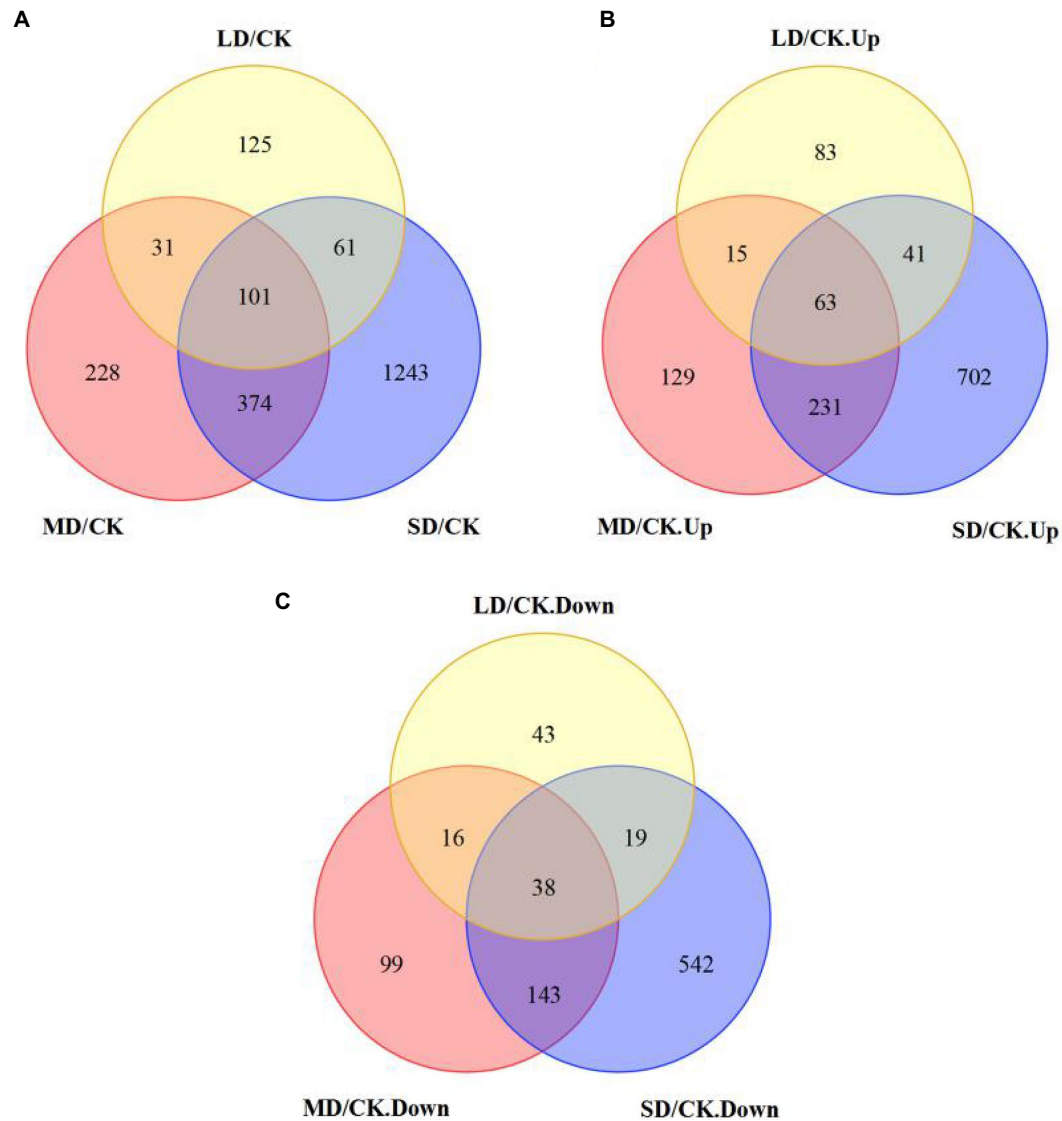
Five differentially expressed genes (DEGs) shown in Venn diagram form. **(A)** Venn diagram showing the total number of total DEGs, **(B)** only upregulated DEGs and **(C)** only downregulated DEGs in *S. davidii* in the LD/CK, MD/CK and SD/CK comparison groups.

A total of 2,163 DEGs (1,264 upregulated and 900 downregulated) were identified in *S. davidii* (Figure 6). For LD/CK, 318 DEGs, including 202 genes that were obviously upregulated and 116 genes that were obviously downregulated, were detected (Figure 6). For MD/CK, 734 DEGs were differentially expressed (438 upregulated and 296 downregulated; Figure 6). For SD/CK, 1779 DEGs were differentially expressed (1,037 upregulated and 742 downregulated; Figure 6). Among these DEGs, only 101 common genes were differentially expressed among all three treatment groups (Figure 6A; Supplementary Table S6). These common DEGs were considered valuable candidate genes for improving drought tolerance.

GO is widely used in gene functional annotation and enrichment analysis. The main purpose for the use of GO in this study was to classify the functions of the predicted *S. davidii* genes. The identified GO terms were associated with three major categories: biological processes, cellular components and molecular functions. In total, 531 of the 734 DEGs in the MD/CK comparison group and 1,245 of the 1,779 DEGs in the SD/CK comparison group were assigned to at least one GO term during drought stress. In the MD/CK comparison group, all significantly enriched DEGs were assigned to biological processes and molecular functions, and 24 and 8 GO terms were identified, among which metabolic process (GO:0008152), organic cyclic compound biosynthetic process (GO:1901362), TF activity, sequence-specific DNA binding (GO:0003700) and nucleic acid-binding TF activity (GO:0001071) were the four most enriched subcategories (Supplementary Table S7). In the SD/CK comparison group, all significantly enriched DEGs were also assigned to biological processes and molecular functions, and 28 and 15 GO terms were identified, among which metabolic process (GO:0008152), regulation of cellular process (GO:0050794), biological regulation (GO:0065007), TF activity, sequence-specific DNA binding (GO:0003700) and nucleic acid-binding TF activity (GO:0001071) were the five most enriched subcategories (Supplementary Table S7). The same enriched GO terms were associated with different functional categories in these two treatment groups, suggesting that these GO terms were involved in the response and tolerance to drought stress and play a vital role in *S. davidii* in response to different degrees of drought stress.

**Figure 7:**
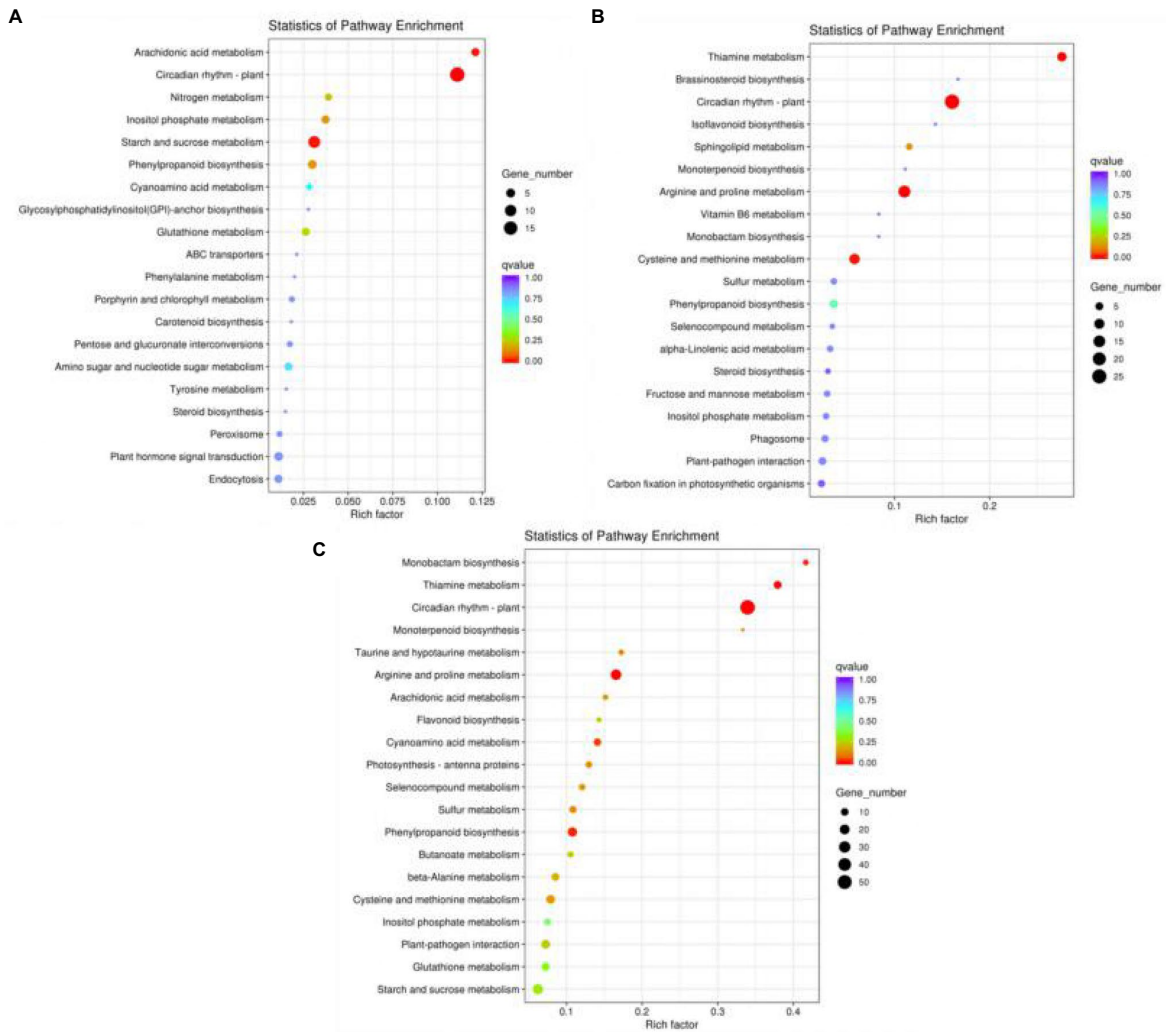
Kyoto Encyclopedia of Genes and Genomes (KEGG) pathway analysis of differentically expressed genes (DEGs). **(A)** Top 20 enriched KEGG pathways in the LD/CK comparison group. **(B)** Top 20 enriched KEGG terms in the MD/CK comparison group. **(C)** Top 20 enriched KEGG terms in the SD/CK comparison group.

KEGG pathway enrichment analysis is an effective method for elucidating the biological functions of DEGs. Therefore, the DEGs enriched in KEGG pathways according to various biological functions were further analysed. DEGs with a *p* ≤ 0.05 were defined as significantly differentially expressed. In our study, among the KEGG enrichment pathways of the LD/CK, MD/CK and SD/CK comparison groups, 7, 6 and 17 significantly enriched pathways (*p* < 0.05), respectively, were identified (Supplementary Table S8). As shown in Figures 7A–C, we screened the top 20 enriched KEGG pathways for the analysis of the LD/CK, MD/CK and SD/CK comparison groups. In the LD/CK comparison group, there were three significantly enriched pathways with a large number of DEGs: circadian rhythm—plant (ko04712, 18 genes), starch and sucrose metabolism (ko00500, 11 genes) and phenylpropanoid biosynthesis (ko00940, five genes; Figure 7A). In the MD/CK comparison group, circadian rhythm—plant (ko04712, 26 genes), arginine and proline metabolism (ko00330, 16 genes), cysteine and methionine metabolism (ko00270, 11 genes), thiamine metabolism (ko00730, eight genes) and phenylpropanoid biosynthesis (ko00940, 6 genes) were the five main pathways (Figure 7B). In the SD/CK comparison group, circadian rhythm—plant (ko04712, 55 genes), arginine and proline metabolism (ko00330, 24 genes), phenylpropanoid biosynthesis (ko00940, 18 genes), cysteine and methionine metabolism (ko00270, 15 genes) and plant–pathogen interaction (ko04626, 15 genes) were the five main pathways (Figure 7C). The KEGG pathway enrichment results thus showed that genes in *S. davidii* were significantly differentially expressed in the LD/CK, MD/CK and SD/CK comparison groups, indicating that the DEGs in these three groups were activated by different molecular mechanisms under drought stress. In this experiment, it was speculated that these pathways might play an important role in the response of *S. davidii* plants to drought stress.

### Changes in TF-Encoding Gene Expression in *S. davidii* Under Drought Stress

**Figure 8:**
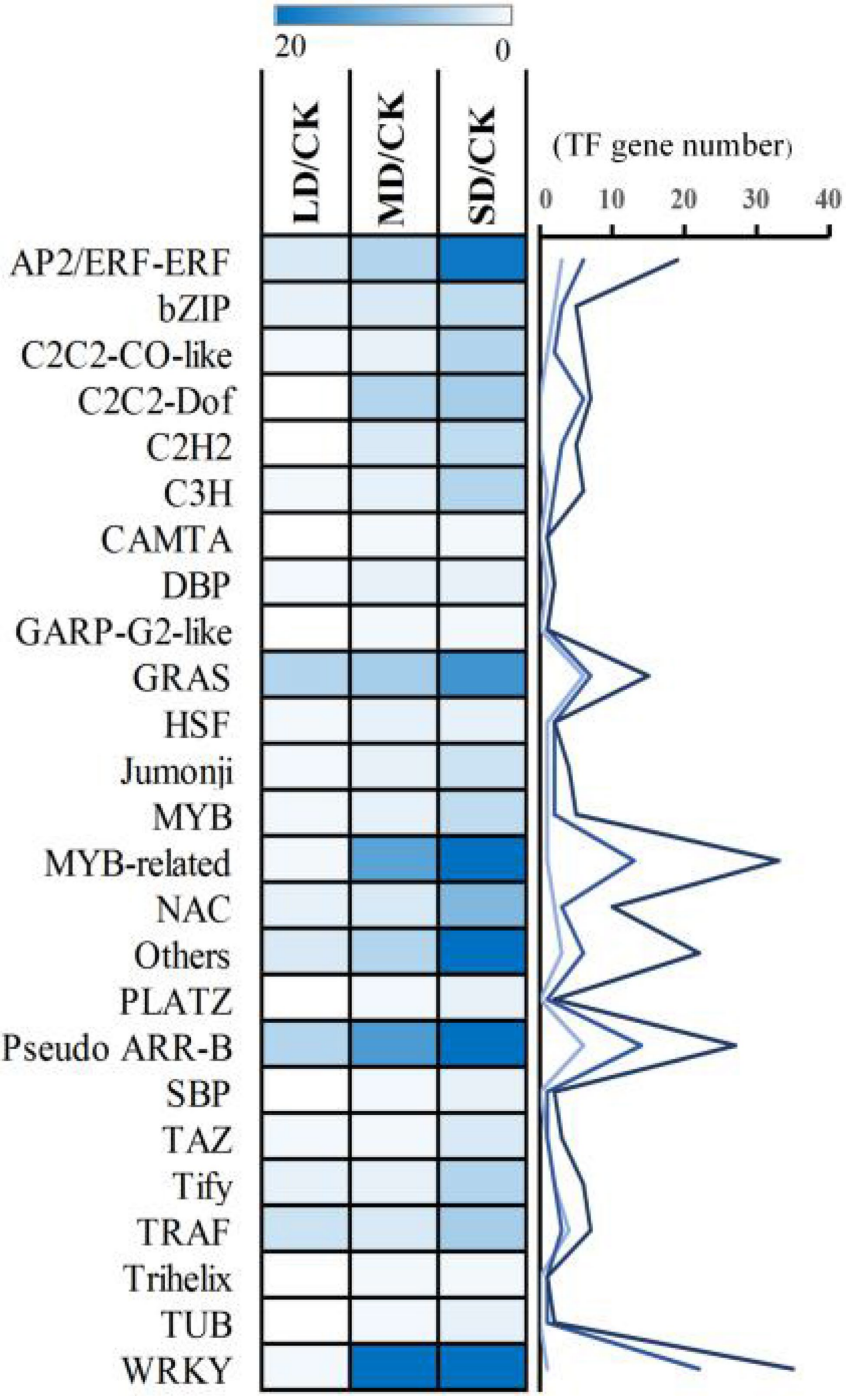
Dynamics of common transcription factor (TF) accumulation profiles.

To analyse the regulatory mechanisms of drought-responsive genes in *S. davidii* in the LD/CK, MD/CK and SD/CK comparison groups, in our transcriptome data, a total of 39, 108 and 257 DEGs were identified as encoding TFs, which could be assigned to 19, 26 and 40 families in LD/CK, MD/CK and SD/CK, respectively (Supplementary Table S9). These TF-encoding genes were classified as members of WRKY, MYB-related, Pseudo-ARR-B, AP2/ERF-ERF, GRAS, NAC, bHLH, C2C2-Dof, TRAF, C2C2-CO-like, C3H, Tify, bZIP, C2H2, MYB and other TF families and subfamilies (Supplementary Table S9). Interestingly, a high percentage of common genes that encode TFs such as WRKY, MYB-related, Pseudo-ARR-B, AP2/ERF-ERF and GRAS TFs was found in the LD/CK, MD/CK and SD/CK comparison groups (Figure 8). These results indicated that these TF-encoding genes might be the main drought tolerance-responsive genes and might play an important role in *S. davidii*.

### Integrative Analysis of Transcriptomics, Metabolomics and Physiological Indices

**Figure 9:**
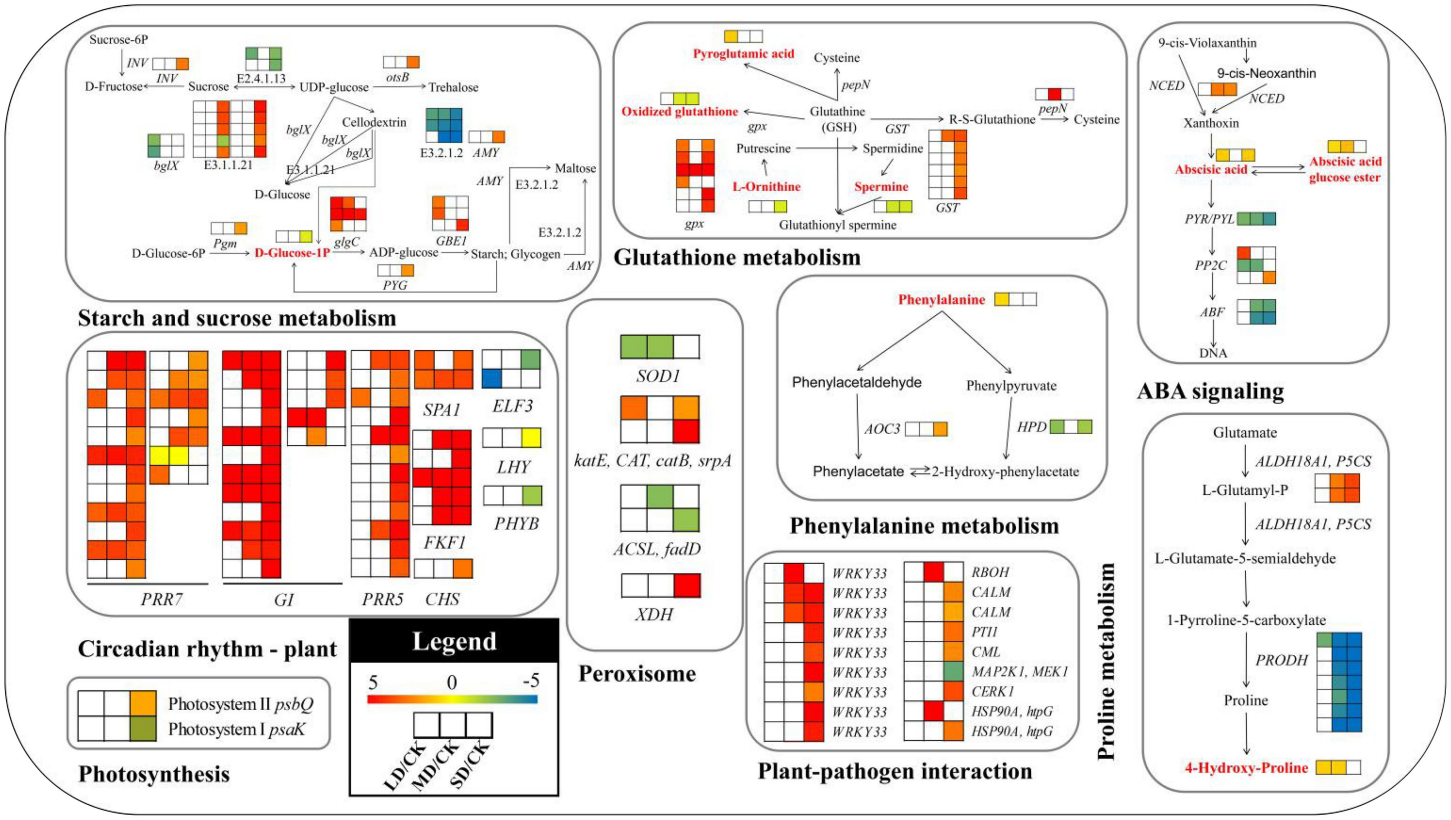
Key metabolic pathways and candidate genes in *S. davidii* associated with the three treatment groups under drought stress. *E3.2.1.2*, beta-amylase; *E2.4.1.13*, sucrose synthase; *E3.1.1.11*, pectinesterase; *E3.2.1.21*, beta-glucosidase; *glgC*, glucose-1-phosphate adenylyltransferase; *GBE1*, 1,4-alpha-glucan branching enzyme; *bglX*, beta-glucosidase; *Pgm*, phosphoglucomutase; *PYG*, glycogen phosphorylase; *INV*, beta-fructofuranosidase; *AMY*, alpha-amylase; *otsB*, trehalose 6-phosphate phosphatase; *gpx*, glutathione peroxidase; *pepN,* aminopeptidase N; *GST*, glutathione S-transferase; *PP2C*, protein phosphatase 2C; *PYL*, abscisic acid receptor PYR/PYL family; *ABF*, ABA-responsive element binding factor; *NCED*, 9-cis-epoxycarotenoid dioxygenase; *PRR7*, pseudoresponse regulator 7; *PRR5*, pseudoresponse regulator 5; *SPA1*, protein suppressor of PHYA-105 1; *ELF3*, protein EARLY FLOWERING 3; *CHS*, chalcone synthase; *FKF1*, flavin-binding kelch repeat F-box protein 1; *GI*, GIGANTEA; *LHY*, MYB-related transcription factor LHY; *PHYB*, phytochrome B; *SOD1*, superoxide dismutase; *katE, CAT, catB, srpA*, catalase; *ACSL, fadD*, long-chain acyl-CoA synthetase; *XDH*, xanthine dehydrogenase/oxidase; *HPD*, 4-hydroxyphenylpyruvate dioxygenase; *AOC3*, primary-amine oxidase; *PRODH*, proline dehydrogenase; *ALDH18A1, P5CS*, delta-1-pyrroline-5-carboxylate synthetase; *HSP90A, htpG*, molecular chaperone HtpG; *WRKY33*, WRKY transcription factor 33; *RBOH*, respiratory burst oxidase; *CALM*, calmodulin; *PTI1*, pto-interacting protein 1; *CML*, calcium-binding protein; *MAP2K1, MEK1*, mitogen-activated protein kinase kinase 1; *CERK1*, chitin elicitor receptor kinase 1; *psbQ*, photosystem II oxygen-evolving enhancer protein 3; and *psaK*, photosystem I subunit X. The log_2_[fold-change (FC)] colour scale ranges from −5 to 5, with blue indicating downregulation and red indicating upregulation (see the colour set scale in the bottom left corner). The red characters represent the metabolites in the pathway diagram.

To screen metabolites and DEGs more accurately, transcriptomic and metabolomic data were further analysed to elucidate the regulatory mechanisms underlying metabolic pathways during drought stress. The relationships between gene expression and many metabolites were determined. A list of detected metabolites, correlated genes and primary metabolic pathways is provided in Figure 9 and Supplementary Table S10. Among the metabolites detected in drought-stressed *S. davidii* plants, **oxidised glutathione** (GSSG) [(downregulated), MD/CK: FC = 0.62; SD/CK: FC = 0.61], **abscisic acid** (ABA) [(upregulated), LD/CK: FC = 1.99; SD/CK: FC = 1.99] and **phenylalanine** [(upregulated), LD/CK: FC = 1.67] had notable regulatory profiles worth highlighting in *S. davidii*. These pathways [glutathione (GSH) metabolism, ABA signalling and phenylalanine metabolism] were also significantly affected by drought stress (Figure 9).

As shown in Figure 9 and Supplementary Table S10, in the ABA signalling pathway, the expression of the gene involved in the synthesis of ABA precursors, 9-cis-epoxycarotenoid dioxygenase (*NCED*), was significantly upregulated in *S. davidii* in the MD/CK and SD/CK comparison groups. Genes encoding ABA receptor PYR/PYL family (*PYL*) members, which are involved in the ABA signal transduction pathway, were downregulated in the LD/CK, MD/CK and SD/CK comparison groups. Among the three different phosphatase 2C (*PP2C*) genes, only one was downregulated in the LD/CK and MD/CK comparison groups. The two ABA-responsive element binding factor (*ABF*) genes were downregulated in both the MD/CK and SD/CK comparison groups. In the circadian rhythm—plant pathway, the pseudoresponse regulator 7 (*PRR7*), pseudoresponse regulator 5 (*PRR5*), protein suppressor of PHYA-105 1 (*SPA1*), chalcone synthase (*CHS*), flavin-binding kelch repeat F-box protein 1 (*FKF1*) and GIGANTEA (*GI*) genes were upregulated in *S. davidii.* However, the EARLY FLOWERING 3 (*ELF3*) and phytochrome B (*PHYB*) genes were downregulated. In the plant–pathogen interaction pathway, all genes (except for *MAP2K1* and *MEK1*, which were downregulated) were upregulated, and WRKY TF 33 (*WRKY33*) genes constituted half of all the genes in this pathway. In the plant starch and sucrose metabolism pathway, the β-amylase (*E3.2.1.2*), sucrose synthase (*E2.4.1.13*) and β-glucosidase (*bglX*) genes were downregulated in *S. davidii.* β-glucosidase (*E3.2.1.21*) genes were significantly enriched in the SD/CK comparison group, and most of them were upregulated. Additionally, the glucose-1-phosphate adenylyltransferase (*glgC*), 1,4-α-glucan branching enzyme (*GBE1*), phosphoglucomutase (*Pgm*), glycogen phosphorylase (*PYG*), β-fructofuranosidase (*INV*), α-amylase (*AMY*) and trehalose 6-phosphate phosphatase (*otsB*) genes were upregulated in *S. davidii*.

Four osmotic regulation-related genes, six oxidative stress-related genes and two photosynthesis-related genes associated with physiological traits were identified in PI401477, and these genes were involved in proline metabolism, phenylalanine metabolism, GSH metabolism, peroxisomes and photosynthesis pathways (Figure 9; Supplementary Table S10). Four osmotic regulation-related genes, the δ-1-pyrroline-5-carboxylate synthetase (*ALDH18A1, P5CS*) and primary-amine oxidase (*AOC3*) genes, were upregulated in the SD/CK comparison group, but the proline dehydrogenase (*PRODH*) and 4-hydroxyphenylpyruvate dioxygenase (*HPD*) genes were downregulated. Six oxidative stress-related genes, including glutathione peroxidase (*gpx*), glutathione S-transferase (*GST*), *CAT* and xanthine dehydrogenase/oxidase (*XDH*), were upregulated only in the SD/CK comparison group, while the *SOD1* gene was downregulated in the LD/CK and MD/SD comparison groups, and long-chain acyl-CoA synthetase (*ACSL*) was downregulated in the MD/CK and SD/CK comparison groups. Two photosynthesis-related genes, photosystem II (PSII) oxygen-evolving enhancer protein 3 (*psbQ*), were upregulated only in the SD/CK comparison group, while the photosystem I (PSI) subunit X (*PsaK*) gene was downregulated.

## Discussion

To adapt to stressful environments in which water is lacking, complex network regulatory mechanisms are activated (Michaletti et al., 2018), changes in gene expression occur and biochemical and molecular processes throughout plants usually occur (Golldack et al., 2014). In this study, in combination with changes in physiological and biochemical indicators, transcriptomic and metabolomic analysis methods were used to identify genes and metabolites that are differentially expressed and differentially accumulate, respectively, under drought stress, and the molecular mechanism of *S. davidii* in response to drought stress was initially elucidated.

Transcriptome (RNA-seq) technology has been widely indicated to be a robust and effective technical method for measuring gene expression during plant development and in response to stress. The application of RNA-seq greatly improves the throughput of individual gene expression profiles of plants under drought stress; when plants with adequate water and plants under drought stress are compared, DEGs can be identified (Cossu et al., 2014). In our study, 101 common DEGs were identified, indicating that these genes play a major role in *S. davidii* plants under drought. The remaining 2062 DEGs accounted for 95.33% of the total DEGs, reflecting the genetic diversity of *S. davidii* plants under varying degrees of drought stress. In this study, the total number of DEGs in the LD/CK and MD/CK comparison groups was much lower than the total number of DEGs in the SD/CK comparison group. However, research by Li et al. (2020) found that the number of genes differentially expressed in tea buds under LD or SD stress was much lower than that under MD stress. Our results showed that SD stress had a more significant impact on the growth of *S. davidii* seedlings, limiting their growth and development. KEGG pathway enrichment analysis based on the DEGs confirmed the differences in the molecular basis underlying the different drought stress responses (Ma et al., 2016).

Studies have found that WRKY (Chen et al., 2017), NAC (Duan et al., 2017) and MYB (Guo et al., 2018) TF-encoding genes play a role in the regulation of drought stress signal transduction pathways. Studies on *Pinus pinaster* have indicated that MYB TFs are related to the energy capture efficiency of the PSII reaction centre (Chen et al., 2014) and are likely to be involved in the systemic response of *P. pinaster* to drought stress (de Miguel et al., 2016). NAC TFs participate in the regulation of the expression of downstream genes involved in the drought stress response of *P. pinaster* (Pascual et al., 2015). Many DEGs that encode TFs were also found in this study, such as MYBs, AP2/ERFs, NACs and WRKYs, indicating that these TFs may be involved in the systemic response of *S. davidii* to drought stress. Additionally, the accumulation of these TFs increased with an increase in the degree of stress, implying that the defence mechanism of *S. davidii* rapidly activates to attenuate drought damage during LD stress and that the plants gradually adapt to the drought conditions during MD and SD stress, showing an ability to rapidly mount defences against and withstand drought.

Metabolic profile analysis can be used to reveal the complex metabolic activity involved in stress adaptation and regulation. Metabolomics studies have been conducted on many plant species under drought stress (Hong et al., 2020; Wan et al., 2021). For example, in tea, 166, 401 and 334 differentially accumulated metabolites were identified in the CK vs. MI, CK vs. MO and CK vs. SE comparison groups, respectively, *via* GC–MS (Li et al., 2020), and a large number of amino acids and their derivatives, organic acids, nucleotides and their derivatives, isoflavones and glycosylflavonoids in response to drought stress were identified. Different types of metabolites accumulate in different plant species in response to different drought stress factors. For example, the amino acid contents in *Pisum sativum* leaves, including proline, valine, threonine, homoserine, inositol, r-aminobutyric acid and trigonelline, significantly increased (Adrian et al., 2008); *Capsicum annuum* leaves accumulate mainly fructose, sucrose, galactinol, cadaverine, putrescine and spermidine (Astrid et al., 2010); and *Trifolium pratense* accumulates mainly rosinol, proline and malic acid (Steven et al., 2014). In the present study, 100, 168 and 281 differentially accumulated metabolites were identified *via* LC–MS in the LD/CK, MD/CK and SD/CK comparison groups, respectively. In addition, several major metabolites were detected in the LD/CK, MD/CK and SD/CK comparison groups. For example, D-serine, N6-acetyl-L-lysine, biochanin A, p-coumaric acid and glycerol chrysin were found to be related to the drought resistance of *S. davidii*.

Combined transcriptomic and metabolomic analysis helps to reveal the complex mechanisms of plants in response to drought at the whole-plant level (Arun et al., 2014). Previous researchers have studied the molecular mechanisms underlying plant drought resistance and have identified candidate genes such as *NCED*, *HY5*, *psbQ*, *GST* and *P5CS*; key metabolites such as phenylalanine, proline and flavonoids; a series of metabolites related to phenylpropanoid biosynthesis, starch and sucrose metabolism and proline; and important biosynthetic pathways and secondary metabolic pathways (Savoi et al., 2016; Dalal et al., 2018; You et al., 2019; Zhao et al., 2019; Min et al., 2020). In this study, we identified several key candidate genes (*NCED*, *LHY*, *gpx*, *GST*, *WRKY33*, *P5CS*, *PRODH*, *AOC3*, *SOD1*, *CAT*, *psbQ*, *psaK*, *glgC*, etc.) and three major metabolites (GSSG, ABA and phenylalanine) involved in GSH metabolism, ABA signal transduction, phenylalanine metabolism, carotenoid biosynthesis, the circadian rhythm-plant pathway, plant–pathogen interactions, starch and sucrose metabolism, proline metabolism and photosynthesis and involved with peroxisomes.

ABA is an inhibitory growth hormone that regulates stress responses and plant growth and development and can promote plant adaptation to drought stress by regulating plant metabolism (Liu et al., 2019; Gai et al., 2020; Nawaz and Wang, 2020). Studies have shown that ABA biosynthesis and signal transduction are closely related to the drought resistance mechanism of plants (Liu et al., 2020). *NCED* is a major rate-limiting factor for ABA synthesis, and its expression is often upregulated under adverse conditions to accelerate ABA synthesis (Pedrosa et al., 2017). The present study found that the *NCED* gene was significantly upregulated under LD and MD stress, and the metabolome data revealed that the accumulation of ABA increased significantly under mild drought and severe drought stress. Therefore, the expression of key genes involved in ABA accumulation and ABA synthesis in *S. davidii* was upregulated under LD and MD stress, implying that ABA induction occurred under LD stress. Thus, ABA has a strong ability to regulate the drought response of *S. davidii*. This study also found that three key genes related to ABA signal transduction were significantly differentially expressed. *PYL* encodes an ABA receptor involved in activating the ABA response (Bai et al., 2019). In the present study, the expression of the *PYL* gene was upregulated under the three different drought stress treatments. Phosphoprotein 2C (*PP2C*) negatively regulates ABA signal transduction (Arshad and Mattsson, 2014). In the present study, it was found that transcripts of this gene increased and decreased among the different treatments. These results were similar to those for *Prunus mongolica* (Wang et al., 2015) under drought conditions. ABFs are TFs of the *bZIP* family and are the main TFs involved in the regulation of the expression of ABA-related genes in plants under abiotic and osmotic stress conditions (Yoshida et al., 2010). In this study, the expression of the *ABF* gene was significantly downregulated under MD and SD stress. Taken together, these results indicate that many ABA signal-related genes are involved in the dual-negative regulatory transduction pathways of ABA signalling, so ABA may play an important regulatory role in *S. davidii* in response to drought stress.

The circadian rhythm is a biological mechanism associated with a temporal cycle formed by plants adapting to the earth’s rotation. The circadian rhythm of plants under abiotic stress changes accordingly in response to external fluctuations, and the optimal occurrence time of important physiological activities of plants adjusts for improved adaptability to adversity (Yerushalmi and Green, 2010; Seo and Mas, 2015). Related studies have verified that the circadian rhythm of many plant species is directly related to drought, and some key drought-related genes and drought-induced physiological activities show differences in circadian expression (Juliana et al., 2014; Kielbowicz-Matuk et al., 2014). In the present study, the plant circadian rhythm pathway (ko04712) was significantly enriched in three different drought treatments, which included 50 key genes (19 *PRR7* genes, 12 *PRR5* genes, 17 *GI* genes and two *ELF3* genes) that were significantly differentially expressed, indicating that drought has a considerable impact on the circadian rhythm of *S. davidii*. Among these genes, *PRR5* and *PRR7* encode components of plant circadian clocks that respond to drought stress and are key genes involved in the plant photoperiod. *PRR7* can regulate a large number of ABA- and drought-responsive genes, and *PRR5* and *PRR7* significantly reduce stomatal conductance under drought conditions (Liu et al., 2013). In addition, transcriptomic and metabolomic data show that genes involved in biosynthetic pathways of compounds such as chlorophyll, carotenoids, ABA, vitamin E and oligosaccharides are controlled by core components of the circadian clocks (PRR5 and PRR7; Fukushima et al., 2009). *GI* and *ELF3* are the key elements regulating the expression of circadian clock genes at night, and they have an important ability to maintain the rhythm of the circadian clock under continuous light conditions (Dixon, 2011). In the present study, the expression of the *PRR7*, *PRR5* and *GI* genes was significantly upregulated under drought conditions, and the expression of the *ELF3* gene was significantly downregulated under LD and SD stress. Based on these results, it is inferred that *PRR7*, *PRR5*, *GI* and *ELF3* may be the key regulators of the *S. davidii* circadian rhythm in response to drought stress and are closely related to light-related signal transduction. Although further research is needed to confirm this hypothesis, our results provide a direction for an in-depth investigation of the regulatory mechanism of the *S. davidii* circadian rhythm in response to drought stress.

Plant–pathogen interactions are rapid disease resistance responses often triggered in plants under abiotic stress. These responses ultimately protect plants from pathogen infection by inducing the expression of defence-related genes (Wu et al., 2014), which constitutes an important defence mechanism (Bonello et al., 2006). In the present study, the plant–pathogen interaction pathways in *S. davidii* seedlings were moderately and severely enriched in DEGs, indicating that the plant–pathogen defence system was rapidly activated. Under abiotic stress, WRKY TFs play an important role in plant transcriptional regulation and pathogen defence (Singh et al., 2017) and have been widely reported in many plant studies (Pandey et al., 2015; Zhang et al., 2018). In the present study, the expression of the *WRKY33* gene was upregulated under MD and SD stress but did not change significantly under LD stress, indicating that *WRKY33* may play a key role in the signal transduction and transcriptional regulation of the *S. davidii* defence response under MD and SD stress. These findings also imply that *S. davidii* employs a rapid defence against pathogen invasion in response to drought stress and adverse conditions.

When plants are drought stressed, photosynthesis is usually adversely affected, resulting in significant downregulated expression of photosynthesis-related proteins such as those involved in the plant photosynthetic system, material synthesis and energy metabolism (Pinheiro and Chaves, 2011; Zhuang et al., 2020). Under SD stress, the expression of a gene encoding a photosystem I (PSI) component, *psaK*, which is involved in electron transport and photosynthetic phosphorylation, was significantly downregulated, which indicated that the light-harvesting capacity, energy transfer and photosynthetic electron transfer of *S. davidii* had been significantly restricted since the beginning of LD stress. At the same time, *psbQ*, a gene related to PSII assembly and stability, was significantly upregulated. Studies have found that a higher RWC plays an important role in maintaining plant chloroplast structure, protecting PSII and improving photosynthetic efficiency (Penella et al., 2014). In the present study, severe drought stress significantly reduced the RWC of *S. davidii*, but it was still remained at a high level, indicating that *S. davidii* has strong water retention capacity and can maintain its chloroplast structure and PSII function under severe drought stress, which significantly and positively affected both PSII structure and stability and ATP synthesis in *S. davidii* during photophosphorylation. The contents of chlorophyll a, chlorophyll b and carotenoids in *S. davidii* leaves increased with a decrease in water potential under stress. These findings are similar to the results of Bahrami et al. (2012) that chlorophyll content in sesame leaves increased first and then remained unchanged under drought stress. These results suggest that the increase in chlorophyll and carotenoid content may be beneficial to protecting the photosynthetic system of *S. davidii* from the effects of short-term drought stress. Starch and sucrose metabolism can cause amylase to hydrolyse starch into glucose, maltose and other oligosaccharides through genes encoding starch-degrading enzymes, thereby maintaining the turgor pressure and water content of leaves under drought stress (Pan et al., 2016; Thalmann et al., 2016). This study showed that SD stress induced significant upregulation of the expression of the α-amylase (*AMY*) gene, while the expression of the β-amylase (*E3.2.1.2*) gene was significantly downregulated under the three drought treatments, indicating that the α-amylase and β-amylase genes work synergistically when plants are drought stressed to promote starch degradation, thereby providing energy for plants to resist drought. In addition, drought stress inhibited the expression of most sucrose-metabolising enzyme-encoding genes. *S. davidii* leaves presented increased expression of 1 β-fructofuranosidase (*INV*) gene during SD stress and decreased expression of 2 sucrose synthase (*E2.4.1.13*) genes under LD and SD stress. Previous studies on the changes in the expression of genes that encode key enzymes involved in the metabolism of insoluble sugars (starch) and soluble sugars (sucrose) in tea plants under drought stress also showed similar results (Yang et al., 2017). The photosynthesis of *S. davidii* gradually decreased, and the amount of products of photosynthesis correspondingly decreased. As such, *S. davidii* obtains energy by breaking down starch and sugar to maintain its physiological metabolism.

Osmotic adjustment is an important means by which plants reduce osmotic potential and resist adverse stress under drought stress. Plants actively accumulate solutes to reduce osmotic potential, thereby reducing water potential and maintaining cell turgor. Osmotic adjustment is achieved by generating large amounts of ions and compatible solutes (such as Pro, SS, SP and organic acids; Farooq et al., 2009). We discovered two pathways related to osmotic regulation: phenylalanine metabolism and proline metabolism. The phenylalanine metabolic pathway is an important secondary metabolic pathway in plants (Sharma et al., 2020). Studies have shown that phenylalanine metabolism is significantly inhibited under drought stress (Gu et al., 2020), and phenylalanine is the precursor of many key secondary metabolic pathways, such as flavonoids and anthocyanins, which affect cell osmotic regulation and improve the drought tolerance of plants (Barchet et al., 2014; Frelin et al., 2017; Lomenova and Hrobonova, 2021). In our study, the expression of the primary-amine oxidase (*AOC3*) gene was upregulated under SD stress and that of the 4-hydroxyphenylpyruvate dioxygenase (*HPD*) gene was downregulated under LD and SD stress. The metabolome study showed that the phenylalanine content increased under LD stress, while the accumulation of p-coumaric acid in phenylpropanoid biosynthesis and the two flavonoid metabolites chrysin and biochanin A increased under the three drought stress treatments. Wang et al. (2019) reported that the aromatic amino acid phenylalanine accumulates significantly in drought-tolerant wild soybeans with consistent results. These results show that the phenylalanine content in *S. davidii* increases under LD stress, thereby promoting the production of flavonoids and improving its drought tolerance. The molecular mechanism of proline metabolism in plant resistance to drought stress has been studied. *P5CS* is a key rate-limiting enzyme involved in the glutamate synthesis pathway of proline, and its activity is feedback inhibited by proline (Meena et al., 2019). Cui et al. (2021) found that *P5CS* was significantly upregulated in *Glycyrrhiza uralensis* under drought conditions. Similarly, Ghaffar et al. (2021) found that *PRODH* was significantly downregulated in *B. napus*. In our study, *P5CS* was significantly upregulated under MD and SD stress, and *PRODH* was significantly downregulated under all three drought treatments. Our study found that the proline and soluble sugar contents in the leaves of *S. davidii* increased significantly with increasing drought stress, while the soluble protein content decreased significantly with increasing drought stress. This result shows that drought stress inhibits protein synthesis and reduces the metabolism of *S. davidii*; however, it is possible that the content of proline and soluble sugar increase to increase the water retention and water absorption capacity of *S. davidii* under drought stress to maintain cell filling, which is a protective response of *S. davidii*. Amino acids play an important role in maintaining intracellular osmotic regulation and the integrity of protein structure. Studies have found that most of the amino acids in the leaves of *B. napus* show a linear increase (Good and Zaplachinski, 2010). In our metabolome study, the two amino acids D-serine and N6-acetyl-L-lysine increased under the three drought stress treatments, indicating that *S. davidii* can adapt to drought stress by increasing the amino acid content.

MDA is the peroxidation product of unsaturated fatty acids in phospholipids, and the level of lipid peroxidation is used as an indicator of free radical damage to cell membranes under stress conditions. Under drought stress conditions, the malondialdehyde and H_2_O_2_ contents in the leaves of *S. davidii* increased with increasing drought stress. Similar results were found for *Spartina alterniflora* (Hessini et al., 2009), indicating that *S. davidii* can be affected by drought stress. The generation of ROS causes membrane lipid peroxidation, which increases the permeability of the cell membrane of *S. davidii*, which in turn leads to cell dysfunction. Under adverse conditions, plants employ an antioxidant enzyme system composed of SOD, CAT and POD to remove excess ROS, thereby avoiding cell membrane damage. In our study, we identified two oxidative stress-related pathways: GSH metabolism and a peroxisome-related pathway. Plant peroxisomes are an important component of the antioxidant system of cells and can eliminate excess ROS produced in plants through a variety of metabolic pathways (Hinojosa et al., 2019). In response to drought stress, the *SOD1* and *CAT* genes involved in the peroxidase pathway were differentially expressed in *S. davidii*; the *SOD1* gene was mildly and moderately downregulated, while the *CAT* gene was mildly and severely upregulated. Ye et al. (2018) found that most *SOD* and *CAT* genes were upregulated in *Hippophae rhamnoides* under drought stress, which contrasted with the results of the present study. In our study, the SOD, POD and CAT activities of *S. davidii* leaves decreased with increasing drought stress, while APX activity showed the opposite trend. Under drought stress, SOD converts toxic substances into H_2_O_2_, which is converted into O_2_ and H_2_O through antioxidant enzymes such as POD and CAT, and excess ROS is eliminated by APX. Drought stress also altered the expression of genes involved in GSH metabolism, in which *GPX*, an antioxidant-related enzyme, reduced the amount H_2_O_2_ and lipid peroxidation by participating in GSH metabolism, which could improve the H_2_O_2_-scavenging ability of plants under abiotic stress to alleviate H_2_O_2_ stress (Noctor et al., 2002). *GST* is a member of a class of antioxidant-related enzymes that can improve the H_2_O_2_-scavenging and cell-detoxifying ability of plants under peroxide stress (Bhabak and Mugesh, 2010). *GST* and *GPX* were found to be significantly upregulated in Chinese pine in response to drought (Zeng et al., 2005), which enhanced plant stress resistance. Consistent with the results of that study, the expression of *GST* and glutathione peroxidase (*GPX*) in the present study was significantly upregulated. Taken together, these results indicated that *GST* and *GPX* played important roles in enhancing the drought tolerance of *S. davidii.* In addition, the metabolome study showed that the oxidised glutathione content decreased in severe drought stress, indicating that drought stress can cause a reduction in oxidised glutathione (GSSG) in plant cells to reduce glutathione (GSH), resulting in a reduction in GSSG content, increasing the GSH/GSSG ratio in plant cells, reducing the lipid peroxidation in plant cells and thereby increasing the resistance of *S. davidii* to drought stress.

**Figure 10:**
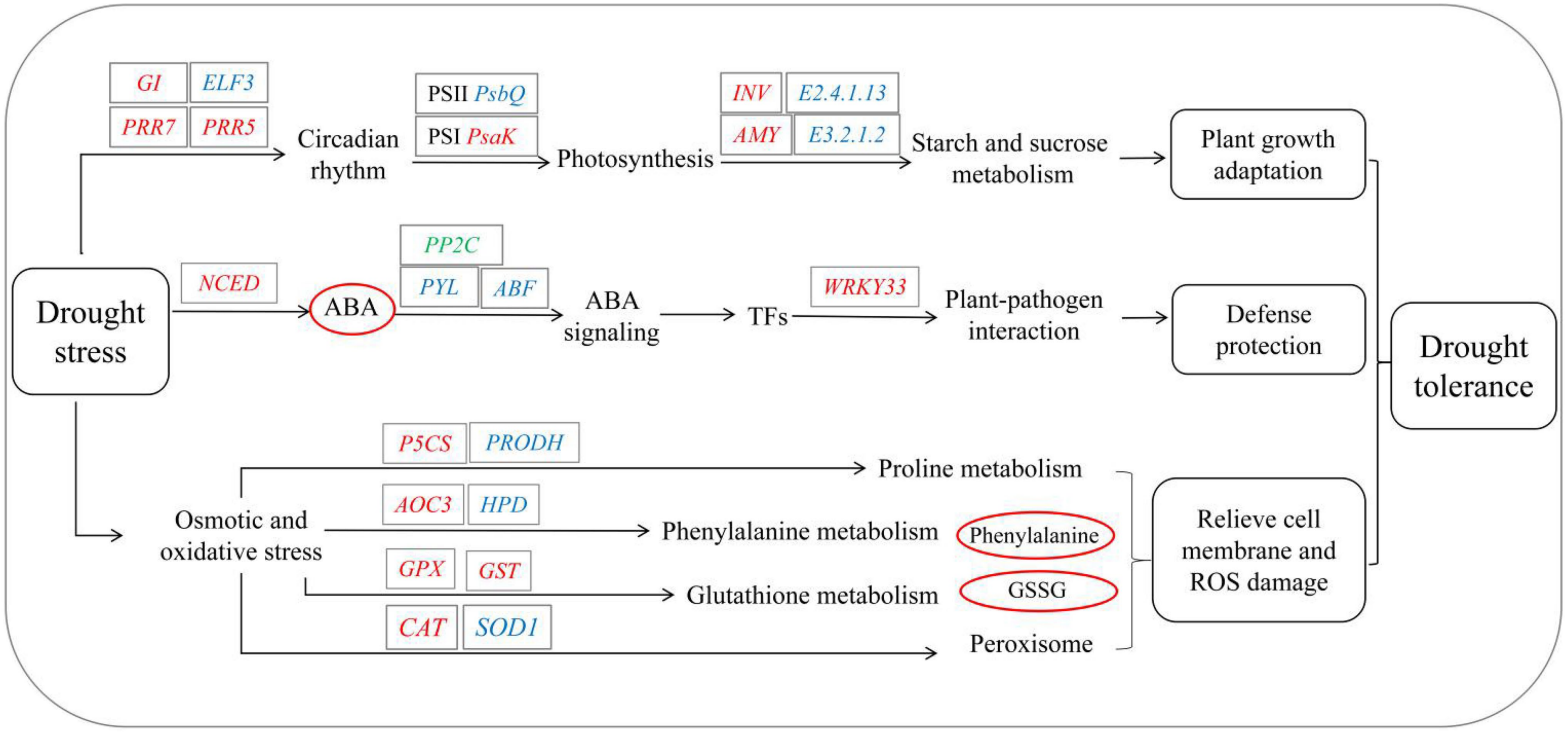
Theoretical molecular mechanism in *S. davidii* in response to drought stress. *E3.2.1.2*, beta-amylase; *E2.4.1.13*, sucrose synthase; *INV*, beta-fructofuranosidase; *AMY*, alpha-amylase; *GPX*, glutathione peroxidase; *NCED*, 9-cis-epoxycarotenoid dioxygenase; *PRR7*, pseudoresponse regulator 7; *PRR5*, pseudoresponse regulator 5; *ELF3*, protein EARLY FLOWERING 3; *GI*, GIGANTEA; *SOD1*, superoxide dismutase; *CAT,* catalase; *PRODH*, proline dehydrogenase; *P5CS*, delta-1-pyrroline-5-carboxylate synthetase; *WRKY33*, WRKY transcription factor 33; *psbQ*, photosystem II oxygen-evolving enhancer protein 3; and *psaK*, photosystem I subunit X. The genes in red, blue or green letters refer to up-, down- and both up- and downregulated expression, respectively, in response to drought. The metabolites in red or blue circles refer to those whose accumulation increased or decreased, respectively, in response to drought.

In summary, the biological systems of *S. davidii* are rebalanced in response to drought stress through the synergistic interaction of various biological processes, such as growth-related, physiological, biochemical processes and molecular responses (Figure 10). On the one hand, the growth of the aboveground parts of *S. davidii* is initially attenuated to adapt to drought stress. The expression of *S. davidii* growth factors decreases, and enzyme activity decreases, weakening the ability of the enzymes to remove ROS. On the other hand, the defence system and protection mechanisms are quickly activated during drought to resist damage. This response manifests as a significant increase in the expression of genes related to ABA biosynthesis and signal transduction, causing rapid accumulation and signal transduction of ABA, inducing the expression of TFs involved in the response to drought stress, altering the circadian rhythm and the metabolism of starch and sugar to promote photomorphogenesis and quickly activating the pathogen defence and protection mechanism to resist pathogen damage. At the same time, *S. davidii* has a strong osmotic adjustment mechanism that helps mitigate the osmotic pressure of cell membranes. Overall, *S. davidii* was found to be strongly drought tolerant by employing a series of strong defence and protection measures to delay and reduce the damage caused by drought.

## Conclusion

To explore the changes in physiological and biochemical characteristics of *S. davidii* under drought stress, key candidate genes and metabolites were assessed. Studies have found that appropriate drought stress can promote an increase in the growth rate, leaf morphology and dry weight of *S. davidii*, while severe drought stress inhibits its growth. To alleviate the damage, *S. davidii* can quickly adjust its physiological and metabolic functions by reducing enzyme activity, increasing osmotic adjustment substances and photosynthetic pigment content, and then adapting to drought conditions. In addition, multilevel analysis was performed on changes in the gene/metabolite expression of *S. davidii*. Drought stress activated 10 important pathways, including ABA signal transduction and its key genes, and increased the accumulation of key metabolites. The integration of transcriptomics and metabolomics data provides theoretical support for further research on the complex mechanisms underlying the drought response of *S. davidii* and other plant species.

## Data Availability Statement

The original contributions presented in the study are publicly available. This data can be found at National Center for Biotechnology Information (NCBI) BioProject database under accession number PRJNA750237.

## Author Contributions

XZ: experimental design, experimental performance, experimental data collection, data analysis, and manuscript writing and revision. L-LZ: seed provision, manuscript writing, resource provision, and funding acquisition. L-JH and X-FS: experimental performance, experimental data collection, and data analysis. P-CW: manuscript writing and revision. All authors contributed to the article and approved the submitted version.

## Funding

This work was funded through projects of the National Natural Science Foundation of China (32060391 and 31702173), Science and Technology Project of Guizhou Province (QKHJC[2020]1Z026 and QKHZC[2021]YB155) and the Guizhou Province Graduate Research Fund (YJSCXJH[2020]039).

## Conflict of Interest

The authors declare that the research was conducted in the absence of any commercial or financial relationships that could be construed as a potential conflict of interest.

## Publisher’s Note

All claims expressed in this article are solely those of the authors and do not necessarily represent those of their affiliated organizations, or those of the publisher, the editors and the reviewers. Any product that may be evaluated in this article, or claim that may be made by its manufacturer, is not guaranteed or endorsed by the publisher.

## Footnotes

^1^ https://www.genome.jp/kegg/pathway.html

^2^ http://www.geneontology.org/

^3^ http://www.genome.jp/kegg/
