# Supplementary material for: Transcriptomic and Metabolomic Analyses Reveal Key Metabolites, Pathways and Candidate Genes in Sophora davidii (Franch.) Skeels Seedlings Under Drought Stress
Source: Front Plant Sci. 2022 Mar 2;13:785702. doi: 10.3389/fpls.2022.785702 (PMC8924449; doi:10.3389/fpls.2022.785702)
Supplement: Supplementary file 1 [file Data_Sheet_1.docx]

**Fig. S1** Morphological characteristics change in *S. davidii* under different drought treatment conditions. Effects of drought stress on **a** growth rate of plant height, **b** dry weight of the aerial part, **c** dry weight of the roots, **d** leaf length, **e** leaf width, and **f** leaf area. The different lowercase letters indicate significant differences (*p* < 0.05) for each index among the different drought treatments.


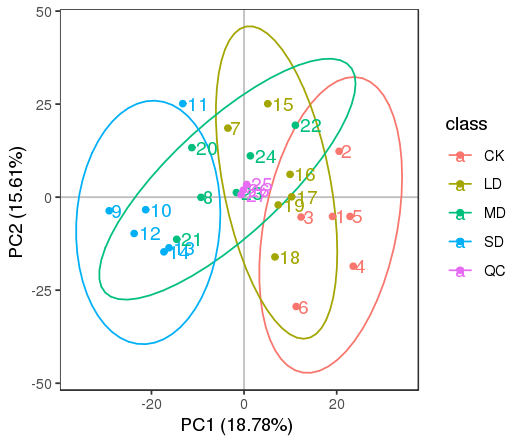


**Fig. S2** PCA of metabolites


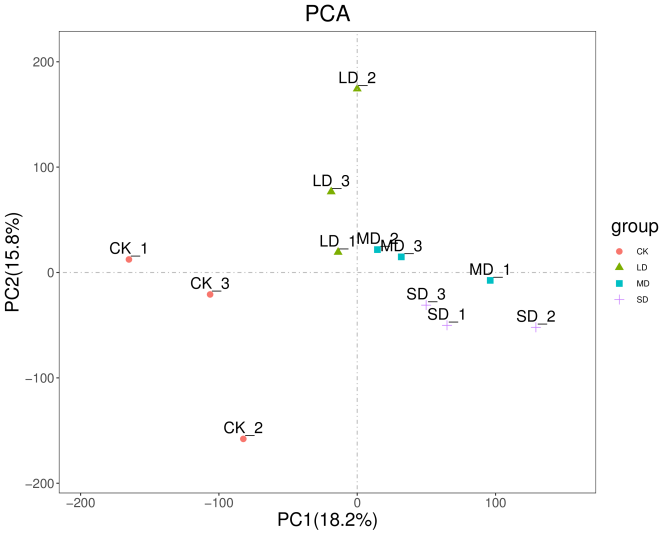

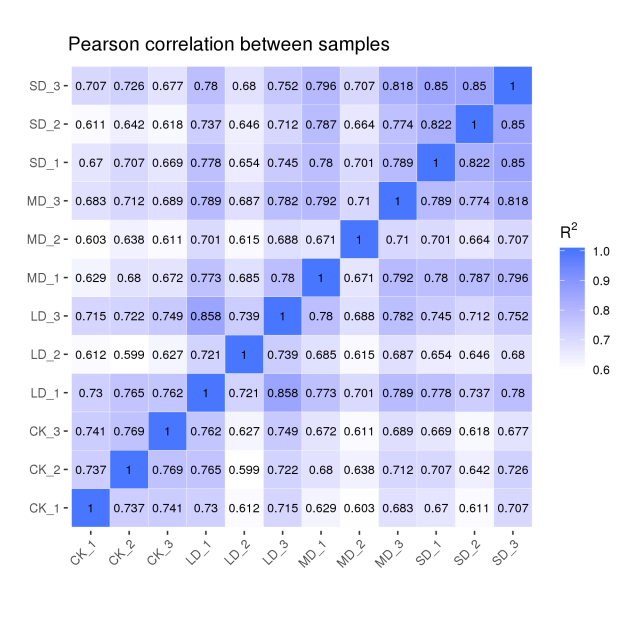


**a**

**b**

**Fig. S3** Transcription profiling. **a** PCCs analysis of the expression patterns of encoding genes under drought stress. **b** PCA analysis of expression genes.
